# Full Crystallographic Imaging of Hexagonal Boron Nitride Monolayers with Phonon‐Enhanced Sum‐Frequency Microscopy

**DOI:** 10.1002/adma.202510124

**Published:** 2025-11-20

**Authors:** Niclas S. Mueller, Alexander P. Fellows, Ben John, Andrew E. Naclerio, Christian Carbogno, Katayoun Gharagozloo‐Hubmann, Damián Baláž, Ryan A. Kowalski, Hendrik H. Heenen, Christoph Scheurer, Karsten Reuter, Joshua D. Caldwell, Martin Wolf, Piran R. Kidambi, Martin Thämer, Alexander Paarmann

**Affiliations:** ^1^ Department of Physical Chemistry Fritz‐Haber‐Institute of the Max‐Planck‐Society 14195 Berlin Germany; ^2^ Department of Physics Freie Universität Berlin 14195 Berlin Germany; ^3^ Department of Chemical and Biomolecular Engineering Vanderbilt University Nashville TN 37235 USA; ^4^ Theory Department Fritz‐Haber‐Institute of the Max‐Planck‐Society 14195 Berlin Germany; ^5^ Department of Mechanical Engineering Vanderbilt University Nashville TN 37235 USA; ^6^ Interdisciplinary Materials Science Program Vanderbilt University Nashville TN 37235 USA; ^7^ Mechanical and Aerospace Engineering University of Florida Gainesville FL 32611 USA

**Keywords:** chemical vapor deposition, heterodyne, hexagonal boron nitride, mid‐infrared, nonlinear microscopy, phonon, sum‐frequency generation

## Abstract

Hexagonal boron nitride (hBN) is an important 2D material for van der Waals heterostructures, single photon emitters, and infrared nanophotonics. The optical characterization of mono‐ and few‐layer samples of hBN however, remains a challenge as the material is almost invisible optically. Here, phase‐resolved sum‐frequency microscopy is introduced as a technique for imaging monolayers of hBN grown by chemical vapor deposition (CVD) and visualizing their crystal orientation. Femtosecond mid‐infrared (IR) and visible laser pulses are used for sum‐frequency generation (SFG), which is imaged in a wide‐field optical microscope. The IR laser resonantly excites a phonon of hBN that leads to an ≈800‐fold enhancement of the SFG intensity, making it possible to image large 100 × 100 µm^2^ sample areas in less than 1 s. Heterodyne detection combined with azimuthal sample rotation further provides full crystallographic information. Combined knowledge of topography and crystal orientation reveals that triangular domains of CVD‐grown monolayer hBN have nitrogen‐terminated zigzag edges. Overall, SFG microscopy is an ultra‐sensitive tool with the potential to image crystal structure, strain, stacking sequences, and twist angles in a wide range of van der Waals structures, where locating and identifying monolayer regions and interfaces with broken inversion symmetry is of paramount importance.

## Graphical abstract

A nonlinear optical microscopy technique is introduced that enables rapid imaging of hexagonal boron nitride monolayers, which are usually optically invisible. The nonlinear mixing of mid‐infrared and visible laser pulses enables full crystallographic imaging through phase‐resolved sum‐frequency generation microscopy, where the resonant excitation of a phonon provides 800‐fold enhancement.

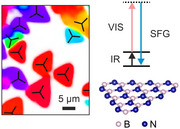

## 1 Introduction

Hexagonal boron nitride (hBN) is one of the most widely used van der Waals (vdW) materials because of its pivotal role as a substrate, encapsulating material, and tunneling barrier in vdW heterostructures.^[^ ^1^, ^2^ ^]^ The material furthermore hosts single photon emitters that can be used as quantum light sources at room temperature.^[^ ^3^, ^4^ ^]^ Moreover, hBN is widely applied for mid‐infrared (IR) nanophotonics because of hyperbolic phonon polaritons that enable nanoscale waveguiding and strong confinement of IR light.^[^ ^3^, ^5^, ^6^, ^7^ ^]^ Besides this pronounced mid‐IR optical response, hBN is optically transparent across the entire near‐IR and visible (VIS) spectral range due to its >5 eV bandgap. This makes it very hard to locate and characterize mono‐ and few‐layers of this material with optical techniques.^[^ ^8^ ^]^ The Raman response of an hBN monolayer, for example, is ≈50 times weaker than that of monolayer graphene, while the more sensitive stimulated and coherent Raman scattering techniques lack crystallographic information for hBN.^[^ ^8^, ^9^, ^10^ ^]^

Fortunately, hBN can be also characterized with 2nd‐order nonlinear techniques, which are sensitive to its crystal structure. This is possible because monolayers and few‐layers with odd layer numbers, as well as twisted interfaces, have broken inversion symmetry.^[^ ^11^, ^12^, ^13^, ^14^ ^]^ Second harmonic generation (SHG) has emerged as a key tool to characterize the crystal orientation and twist angles of many 2D vdW materials.^[^ ^15^, ^16^, ^17^, ^18^, ^19^, ^20^ ^]^ Due to the selection rules of SHG, a characterization of the polarization dependence gives access to the crystallographic axes.^[^ ^11^ ^]^ This method has also been recently used to image the twist angles and stacking configurations of buried interfaces between hBN films.^[^ ^13^ ^]^ There are, however, two challenges that prevent SHG from becoming a standard tool for hBN characterization: 1) The SHG intensity from hBN mono‐ and few‐layers is ≈1000 times weaker than that of transition metal dichalcogenide (TMD) monolayers.^[^ ^11^ ^]^ This is because hBN is lacking optical resonances in the VIS and near‐IR, while TMDs have excitonic resonances that enhance the SHG response. 2) An SHG intensity measurement does not provide full crystallographic information, i.e., it detects a 6‐fold symmetry, whereas the lattice of hBN monolayers has 3‐fold symmetry, and therefore cannot distinguish B‐N from N‐B crystal directions.^[^ ^11^ ^]^

Here, we establish sum‐frequency generation (SFG) microscopy for addressing these two challenges. We use our recently developed wide‐field optical microscopy (Refs.[21, 22]) to image SFG signals generated from the combined illumination of the sample with fs laser pulses in the mid‐IR and VIS spectral ranges. The mid‐IR laser covers a phonon resonance of hBN monolayers with the bandwidth of the IR pulse, which leads to an ≈800‐fold intensity enhancement, making SFG equally efficient as SHG in excitonic TMDs. This enables rapid screening of substrates for hBN monolayers. Additionally, by heterodyning the SFG signal with a local oscillator and using balanced imaging,^[^ ^21^ ^]^ we measure the amplitude and phase of the SFG signal with high sensitivity, giving access to the full crystal orientation. We demonstrate this by imaging hBN monolayer islands on a transparent fused silica substrate that were grown by chemical vapor deposition (CVD) on a catalytic substrate before transfer.^[^ ^23^, ^24^ ^]^ By analyzing the SFG signal when azimuthally rotating the sample, we image the topography and crystal orientation simultaneously and find that the hBN islands have nitrogen‐terminated zigzag edges. The technique of SFG microscopy is applicable to a wide range of vdW heterostructures containing hBN and may be used to image strain, stacking order, and twist angles in the future.

## 2 Results

### 2.1 Phase‐Resolved Sum‐Frequency Microscopy

**Figure 1:**
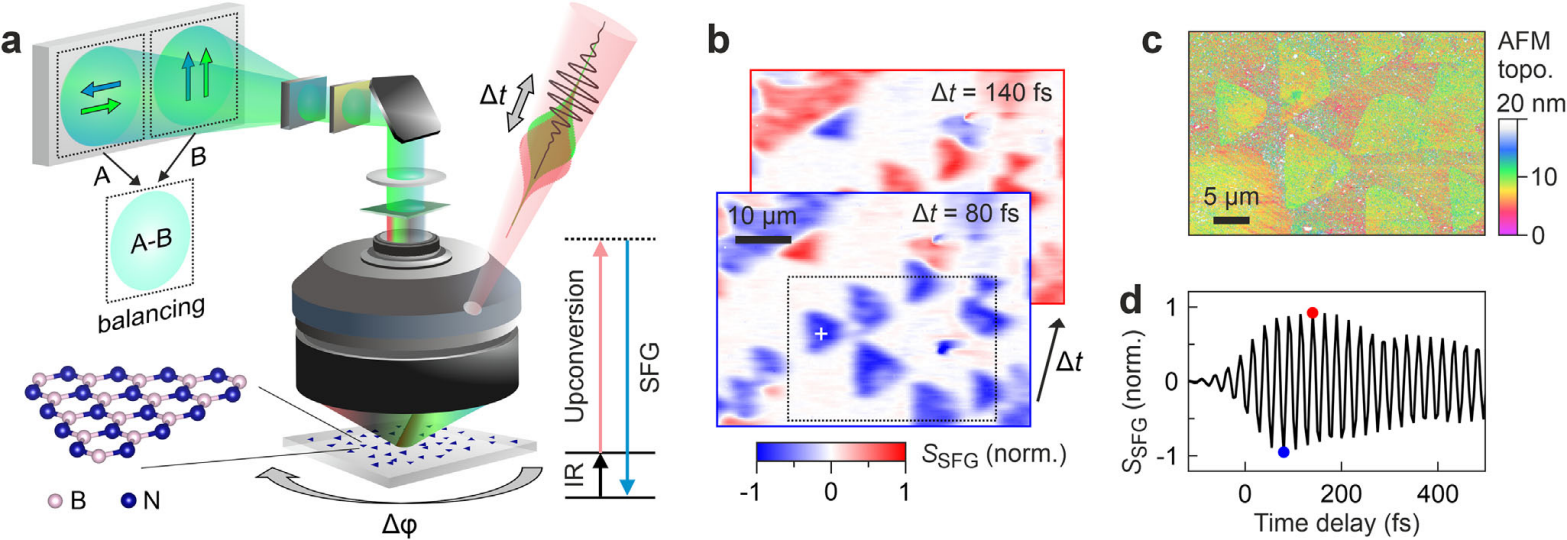
Phase‐Resolved SFG Microscopy of hBN. a) Schematic of the SFG microscope. An IR beam (black) and VIS upconversion beam (red) with controllable time delay Δ*t* collinearly illuminate a large sample area (150 × 200 µm) through a hole in a reflective objective. The nonlinear SFG signal (blue) is collected by the objective and guided through filters to a CCD camera. The SFG light is interfered with a collinear local oscillator (green) for varying time delays to obtain phase and spectral information. Two interference images with orthogonal polarization components (A and B) are recorded and balanced (A‐B) to measure the SFG amplitude. The sample can be azimuthally rotated by Δ*φ* (Video S1, Supporting Information). The inset shows a sketch of the hBN crystal structure. b) SFG amplitude images of hBN monolayer flakes grown by CVD recorded for two time delays Δ*t*, indicated by red and blue dots in (d). c) AFM topography image of the area in (b) that is highlighted with a dotted box. d) SFG amplitude as a function of IR‐VIS time delay Δ*t*, evaluated at a single pixel shown as a white cross in (b).

The hBN monolayers are imaged and characterized with phase‐resolved SFG microscopy (**Figure** **1**a, Experimental Section, refs. [21, 22, 25]). An ultrafast laser is used to generate two broadband beams: one in the mid‐IR (≈7 µm, ≈100 fs, ≈5 µJ, *p*‐polarization) and a second in the visible frequency range for upconversion (≈690 nm, ≈100 fs, ≈3 µJ, *p*‐polarization). The two beams are then combined and collinearly directed through a custom‐made hole in a reflective Schwarzschild objective to illuminate the entire field‐of‐view.^[^ ^21^ ^]^ The two laser pulses generate an SFG signal at the sample that is imaged with a CCD camera. In order to obtain phase information, a local oscillator (LO) beam that has the same frequency as the SFG signal is further guided collinearly into the microscope.^[^ ^21^, ^26^, ^27^ ^]^ The reflected LO then interferes with the SFG signal from the sample, with the interference term being isolated through paired‐pixel balanced imaging in order to extract a signal *S*_SFG_ proportional to the SFG amplitude (Experimental Section).^[^ ^21^ ^]^ For this, two images are simultaneously generated on the CCD camera that have opposite signs of the interference cross term of SFG and LO. Subtracting the two images and referencing yields a balanced image of *S*_SFG_ with strongly reduced noise that contains phase information (Figure 1b).

We use the SFG microscope to characterize monolayers of hBN that were grown by CVD on an Fe catalyst.^[^ ^23^, ^24^, ^28^ ^]^ (Figure 1b–d). The CVD growth predominantly resulted in triangular monolayer domains, while multilayers formed only at a few locations with smaller sizes (Experimental Section, Figure S7, Supporting Information). After transfer to a transparent fused silica substrate the monolayers are optically undetectable in a standard linear reflection microscope (Figure S4a, Supporting Information) because of the ≈3.3 Å thickness of the monolayers and no optical resonances of hBN in the visible spectral range.^[^ ^8^ ^]^ In contrast, the SFG microscope enables imaging of hBN monolayers across a large (≈200 × 150 µm^2^) sample area in <1 s (Figure 1b; Figure S4b, Supporting Information). The images clearly resolve the triangular shape of the hBN monolayer islands as the spatial resolution (≈1 µm here) is limited by the visible SFG wavelength, which is much shorter than the mid‐IR diffraction limit at λ_IR_≈7 µm.^[^ ^21^, ^22^, ^29^ ^]^ A comparison to an AFM topography image of a selected smaller area (Figure 1c, >30 min acquisition time) demonstrates the high chemical selectivity of SFG microscopy to the hBN monolayers, whereas the AFM image is also affected by contamination and surface roughness, which strongly reduces the contrast. The different signs and magnitudes of *S*_SFG_ from the hBN islands already hint to different crystal orientations, as shown below.

To obtain spectral information, an interferogram is recorded for each image pixel by scanning a delay of the mid‐IR pulse with respect to the VIS and LO pulses (Figure 1b,d; Video S2, Supporting Information).^[^ ^26^, ^30^ ^]^ As such, the technique can be understood as a pixel‐wise, nonlinear‐optical version of Fourier‐transform IR (FTIR) spectroscopy, but additionally providing phase information. The SFG amplitude directly probes the IR field‐resolved optical response of the material, which here is dominated by the ringing of the transverse optical IR phonon resonance of hBN (Figure 1d). A similar response was also recently probed by phonon‐enhanced four‐wave mixing.^[^ ^31^ ^]^ The spectral response can then be obtained from a Fourier transformation (see below). As this spectral response is probed simultaneously for the entire wide‐field microscope image, the SFG microscope enables a direct correlation of spatial and spectral information through hyperspectral imaging.

### 2.2 Phonon‐Enhanced Sum‐Frequency Generation

**Figure 2:**
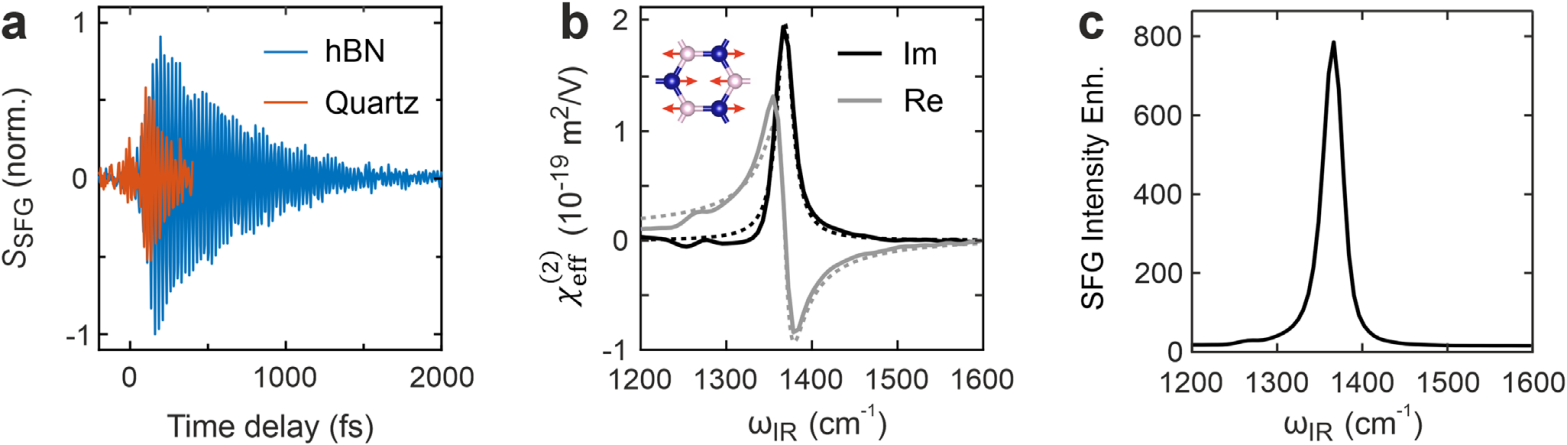
Phonon‐Enhancement of SFG. a) Time‐domain interferogram of SFG amplitude for monolayer hBN (blue, evaluated at white cross in Figure 1b) and *z*‐cut α‐quartz (orange) by scanning a time delay of the mid‐IR pulse with respect to the VIS and LO pulses. b) Measured SFG spectrum (solid lines) of monolayer hBN with the E’ TO phonon resonance at *ω*_TO_ = 1368 cm^−1^ (see inset). A fit with Equation (1) is shown as dashed lines. c) Enhancement of the SFG intensity by the IR phonon resonance of hBN. The resonant $|\chi_{\mathrm{eff}}^{\left(2\right)}\left(\omega_{\mathrm{IR}}\right){|}^{2}$ was referenced by the off‐resonant $|\chi_{\mathrm{eff}}^{\left(2\right)}{|}^{2}$ measured in Ref. [11].

The IR phonon resonance of hBN at ≈7.3 µm leads to a strong enhancement of the SFG signal (**Figure** **2**). We measure an integrated SFG response of hBN monolayers that is ≈10x larger than the off‐resonant response of a *z*‐cut α‐quartz substrate that is used as a phase and amplitude reference (Figure 2a). This is remarkable as bulk quartz has broken inversion symmetry, leading to an effective SFG probing depth (coherence length) of ≈36 nm, which is ≈100x thicker than the hBN monolayer (Section S4, Supporting Information). After a Fourier transformation and referencing with quartz,^[^ ^30^ ^]^ we obtain the spectrum of the effective nonlinear susceptibility of monolayer hBN, which shows a pronounced resonance from the *E*’ transverse optical (TO) phonon of hBN at ω_TO_ = 1368 cm^−1^ (Figure 2b). The spectral dependence is well explained by (see Section S1 Supporting Information for full tensorial expression)^[^ ^32^, ^33^ ^]^

(1) $$\chi^{\left(2\right)}\left(\omega_{\mathrm{IR}}\right)=\chi_{\infty }^{\left(2\right)}\left(1+\frac{\alpha_{R}Z^{*}}{2V_{\mathrm{uc}}M}\frac{1}{\omega_{\mathrm{TO}}^{2}-\omega_{\mathrm{IR}}^{2}-i\gamma_{\mathrm{TO}}\omega_{\mathrm{IR}}}\right)$$

where $\chi_{\infty }^{\left(2\right)}$ is the off‐resonant susceptibility from electronic transitions at larger frequencies, *ω*_IR_ the IR laser frequency, *γ*_TO_ the damping of the phonon, *V*_UC_ the unit cell volume, *M* the effective mass, *α*_R_ the Raman polarizability, and *Z* ^*^ the Born effective charge (Figure 2b, dotted lines). This shows that the phonon needs to be both Raman‐ and IR‐active in order to enhance the SFG response, which is generally the case for materials with broken inversion symmetry, like hBN monolayers.^[^ ^32^, ^33^, ^34^ ^]^ The phonon‐enhanced *χ* ^(2)^ is particularly large in hBN monolayers because of the strongly anharmonic potential associated with the atomic vibrations.^[^ ^35^ ^]^ On resonance, we obtain an effective surface susceptibility of $\chi_{\mathrm{eff}}^{\left(2\right)}$(*ω*_TO_) = 1.9 × 10^−19^ m^2^ V^−1^, which corresponds to a bulk susceptibility of *χ* ^(2)^(*ω*_TO_) ≈ 580 pm V^−1^ when assuming a thickness of *d*_hBN_ = 0.33 nm for monolayer hBN. This is much larger than the off‐resonant susceptibility $\chi_{\infty }^{\left(2\right)}$ = 20.8 pm V^−1^ measured previously by SHG on hBN monolayers using near‐IR lasers.^[^ ^11^ ^]^ Comparing to this off‐resonant response, we find a ≈790‐fold enhancement of the SFG intensity by the IR phonon resonance (Figure 2c; Section S4, Supporting Information). This makes SFG of hBN monolayers equally efficient as SHG of transition metal dichalcogenides, which have excitonic resonances in the visible spectral range, and where SHG emerged as a standard tool to characterize crystal orientations and twist angles.^[^ ^15^, ^16^, ^18^, ^19^, ^20^, ^36^ ^]^

### 2.3 Full Crystallographic Imaging of hBN Monolayers

**Figure 3:**
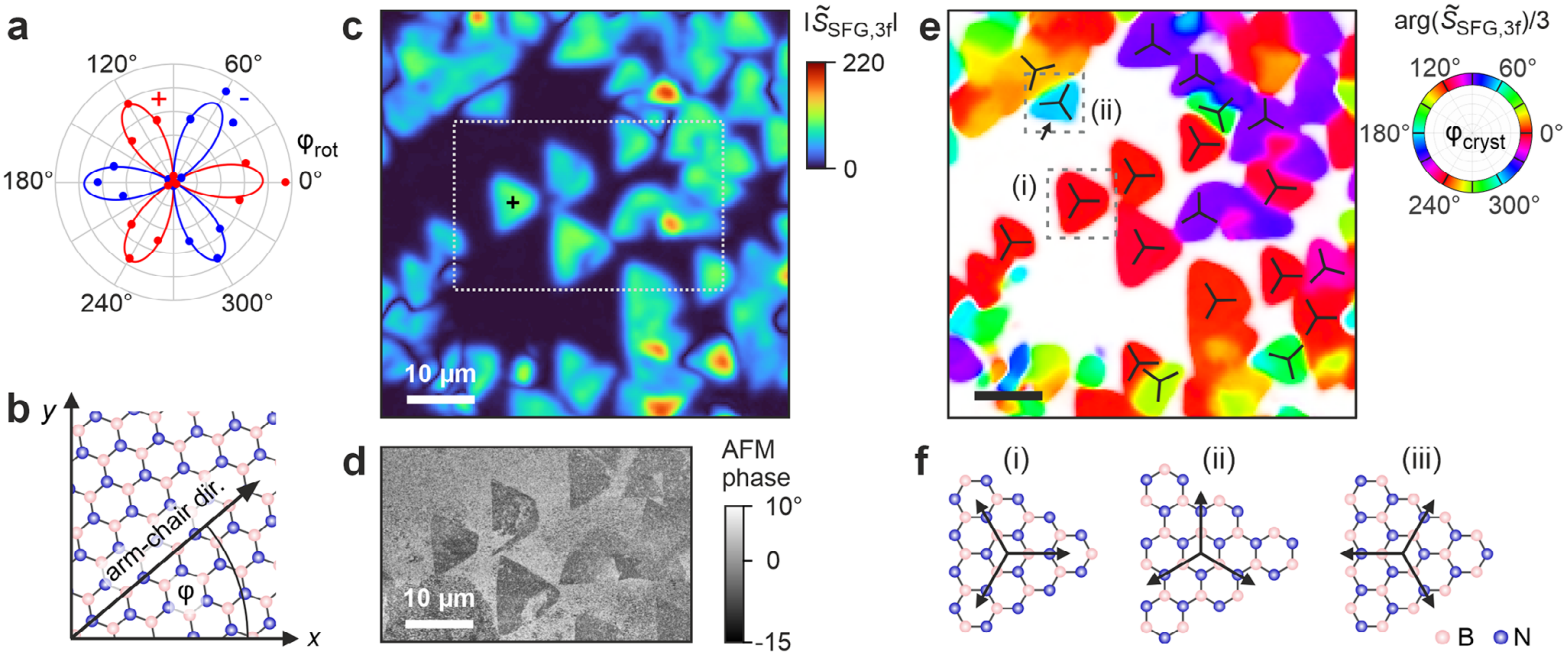
Crystallographic Imaging of hBN Monolayers. a) Resonant SFG amplitude Im[*S*_SFG_(*ω*_IR_ = *ω*_TO_)] of monolayer hBN [cross in (c)] as a function of azimuthal sample rotation angle *φ*_rot_, with red indicating positive and blue negative sign. b) Angle *φ* = *φ*_cryst_ – *φ*_rot_ between the crystallographic B‐N arm‐chair direction and in‐plane polarization of all beams along *x*. c) Image of SFG amplitude |$\tilde{S}$_SFG,3f_| with 3‐fold rotational symmetry obtained from pixel‐wise Fourier analysis of Im[*S*_SFG_(*φ*_rot_)] at *ω*_IR_ = 1368 cm^−1^ (see Figure S5 Supporting Information for full measurement area). d) AFM phase image of the area in (c) that is highlighted with a gray dotted box. Compared to an AFM topography image of the same area in Figure 1c the AFM phase image is more sensitive to changes in composition and layer number. e) Image of rotational phase arg($\tilde{S}$_SFG,3f_)/3 = *φ*_cryst_ of the SFG amplitude with 3‐fold rotational symmetry obtained with a similar analysis as for (c). Black lines show the arm‐chair crystal directions and colors the angle *φ*_cryst_ between the arm‐chair direction and *x‐*axis. f) Orientation of B‐N armchair direction (arrows) with respect to triangle edges depending on edge termination, with i) N‐terminated zigzag edges, ii) armchair edges, and iii) B‐terminated zigzag edges. Dashed boxes in (e) show flakes with i) three N‐terminated zigzag edges and ii) two N‐terminated zigzag and one armchair edge (see arrow), while B‐terminated zigzag edges are not observed in our sample.

Beyond visualization of hBN monolayers, phase‐resolved SFG microscopy gives access to their full crystallographic orientation (**Figure** **3**). When rotating the sample azimuthally with respect to the laser polarization, the resonant SFG amplitude at *ω*_IR_ = *ω*_TO_ oscillates and follows the 3‐fold symmetry of the D_3h_ crystal lattice of monolayer hBN, with *S*_SFG,_ *_x_* ∝ cos(3*φ*), where *φ* is the angle between the arm‐chair crystal direction and the in‐plane component of the laser polarization (Figure 3a; Sections S1–S3, Supporting Information). The SFG signal is thus largest when the laser beams are polarized along the armchair crystal directions, and possesses a positive sign along the B‐N direction vs a negative sign along N‐B (Figure 3a,b). In contrast to previous SHG intensity measurements, which detected a 6‐fold symmetry |cos(3*φ*)|^2^,^[^ ^11^, ^12^, ^13^, ^14^ ^]^ the heterodyned measurements here allow us to determine the full crystal orientation.^[^ ^17^ ^]^

In order to visualize the topography and crystal orientation of all hBN islands, we use a rotational Fourier analysis of the SFG images recorded for different azimuthal sample rotations (Figure 3c,e; Experimental Section; Supporting Information).^[^ ^22^, ^25^ ^]^ For each image pixel, this approach extracts the experimental SFG signal component $\tilde{S}$_SFG,3f_ with 3‐fold symmetry, and thus probes the D_3h_ crystal symmetry of hBN. The Fourier analysis provides both a magnitude image |$\tilde{S}$_SFG,3f_| that shows the topography (Figure 3c) and a rotational phase image arg($\tilde{S}$_SFG,3f_)/3 that shows the crystal orientation of all hBN islands (Figure 3e). In contrast to a single sample rotation, |$\tilde{S}$_SFG,3f_| visualizes all hBN islands independent of their individual crystal orientation (compare Figures 1b and 3c). The SFG image thus provides detailed information about the shape and structure of the hBN islands, and correlates well with the topography probed in an AFM phase measurement of a selected smaller sample area, even resolving small morphological imperfections (compare Figure 3c dashed box with Figure 3d).

The rotational phase image directly visualizes the local crystal orientation of the hBN monolayer islands, which were grown into a partially continuous film by CVD (Figure 3e). Most of the individual islands are single crystals and have triangular shapes, which enables a correlation of their shape with the crystal orientation. Analysis of these images shows that for almost all islands, the armchair crystal directions point toward the corners of the triangles, which implies that the islands have zigzag edges [Figure 3f(i),e black lines]. We also observe a few flakes with irregular shapes that have arm‐chair edges, Figure 3e,f(ii). The prevalence of zigzag edges was also suggested by CVD growth models, which revealed that zigzag edges are energetically more favorable.^[^ ^23^, ^37^, ^38^ ^]^ Phase‐resolved SFG enables us to distinguish islands with opposite (180° rotated) crystal orientations, which would have identical signal in an intensity measurement. Through comparison with density functional theory calculations (Section S2, Supporting Information), we expect a positive SFG amplitude when the B‐N armchair crystal direction points along the positive *x*‐axis (Figure 3a,b, *φ* = 0). This suggests that the vast majority of hBN islands have N‐terminated zigzag edges, Figure 3e,f (i), whereas we do not observe B‐terminated edges, Figure 3f(iii). This was indeed also concluded from microscopic theory, suggesting that N‐terminated zigzag edges are energetically more favorable than their B‐terminated counterpart.^[^ ^38^ ^]^ Furthermore, N‐terminated zigzag edges were primarily observed in local measurements with STM and TEM.^[^ ^39^, ^40^ ^]^ Here, phase‐resolved SFG microscopy with azimuthal scanning enables us to measure the edge termination across much larger length scales, as well as the simultaneous characterization of many hBN islands.

Several areas in the SFG magnitude image appear brighter than the hBN monolayer domains (Figure 3c; Figure S5, Supporting Information). These areas can be identified as few‐layer hBN through a comparison with AFM phase images (Figure S6, Supporting Information). The increased SFG signal is however surprising as bi‐ and few‐layers with standard AA’ stacking have inversion symmetry and should have no second‐order nonlinear response, whereas few‐layers with odd layer number should have the same SFG signal as monolayers.^[^ ^11^, ^13^ ^]^ Instead, we observe approximately a factor × 2 increase in SFG signal for these domains (Figure S5, Supporting Information). This suggests that these layers grow with non‐standard stacking configurations, such as AB or AA stacked bilayers, for which a × 2‐fold increase in second‐order nonlinear signal is expected.^[^ ^41^ ^]^

## 3 Discussion

Overall, we have shown that phase‐resolved SFG microscopy in combination with azimuthal scanning enables full crystallographic imaging of hBN monolayers with IR‐subdiffractional spatial resolution. Compared to SHG, SFG is strongly enhanced by matching the IR laser wavelength with a phonon resonance of the material that is both IR and Raman‐active. This makes SFG of hBN monolayers equally efficient as SHG of excitonic 2D materials and thereby enables rapid screening of this usually invisible layer, with sensitivity to crystal orientation and more generally also layer number and stacking configuration. The technique further naturally provides images across a range of IR frequencies that enable chemical selectivity and allow to distinguish different materials. SFG microscopy may thus also be used to image strain distributions,^[^ ^42^ ^]^ with combined sensitivity to magnitude and direction by probing phonon frequency shifts and polarization selection rules, as well as to image frequency‐dependent propagation of polaritons.^[^ ^34^, ^43^ ^]^ As a far‐field optical technique that is selective to hBN through its IR phonon resonance, SFG microscopy has the potential to image spatial heterogeneity and twist angles of hBN inside of van der Waals heterostructures and devices in a non‐invasive and label‐free way. More generally, the technique is also applicable to a wide range of other van der Waals materials with broken inversion symmetry, interfaces between materials, as well as combinations with anisotropic molecular assemblies.

Furthermore, the large phonon‐enhanced second‐order nonlinearity of monolayer hBN is particularly appealing for converting mid‐IR to visible light, which could enable new optoelectronic devices and detectors based on vdW heterostructures. The conversion efficiency could be even further increased by using 3R‐stacked hBN,^[^ ^44^ ^]^ where the nonlinear intensity is expected to increase quadratically with layer number,^[^ ^45^ ^]^ and by nanophotonic structures,^[^ ^34^, ^46^ ^]^ potentially enabling continuous‐wave upconversion or visible detection of thermal radiation.^[^ ^47^, ^48^ ^]^ The sensitivity to stacking order makes SFG microscopy also promising for rapid screening and in situ imaging of sliding ferroelectricity, where the inversion symmetry of hBN bi‐ and multilayers is broken through their local stacking reconfiguration.^[^ ^44^, ^49^, ^50^ ^]^ The nonlinear response of hBN can be further tailored through nonlinear phonon‐mediated processes that are expected at large IR laser intensities, such as polariton blockade in nanometer‐sized structures and strong frequency shifts by mixing static and optical fields.^[^ ^35^ ^]^ Such phonon‐driven effects, also including helical and chiral nonlinear phononics,^[^ ^51^ ^]^ could be characterized with pump‐probe measurements, which are compatible with our SFG time‐domain approach.^[^ ^52^ ^]^

## 4 Experimental Section

### Phase‐Resolved SFG Microscopy

The details of the azimuthal‐scanning, phase‐resolved SFG microscope used in this work could be found elsewhere in previous works.^[^ ^21^, ^22^, ^25^, ^26^ ^]^ In short, the ≈30 fs, ≈800 nm output of a 1 kHz Ti: sapphire laser (≈7 W, Coherent Astrella) was split in two, with each beam seeding two independent optical parametric amplifiers (OPA, Light Conversion Topas Prime), see Figure S8 (Supporting Information). The signal and idler of the first OPA were then combined in a nonlinear crystal to produce their tunable difference‐frequency (DFG) output in the mid‐IR, and the signal of the second OPA was frequency‐doubled to produce a 690 nm (VIS) beam. The mid‐IR beam was then split by a 45° (s‐polarized) KBr beam splitter, with the weaker part being combined collinearly with the VIS and directed through a *z*‐cut α‐quartz window for LO generation. The transmitted VIS and output LO beams then passed by a delay stage and are subsequently combined collinearly with the stronger part of the mid‐IR and sent to the microscope. The setup is sketched in Figure S8 (Supporting Information).

In the microscope setup, a single collinear beam comprising mid‐IR (p‐polarized), VIS (p‐polarized), and LO (s‐polarized) pulses was focused onto the sample at an incidence angle of 36° relative to the surface normal using a parabolic mirror. The beams were directed through a custom‐made hole in a reflective Schwarzschild objective (40x, PIKE Technologies, 0.78 NA). The SFG and reflected light were collected by the objective, filtered to remove all frequencies except the SFG (and LO), and focused onto a thermoelectrically cooled EMCCD camera. To implement balanced imaging,^[^ ^21^ ^]^ before the camera, the SFG and LO polarizations were rotated by 45° and then split by a polarizing beam splitter to produce two images with opposing interference (±), with intensity

(2) $$I_{\mathrm{het}}\left(\Delta t\right)\propto {\left|E_{\mathrm{SFG}}\left(\Delta t\right)\right|}^{2}+{\left|E_{\mathrm{LO}}\right|}^{2}\pm 2E_{\mathrm{SFG}}\left(\Delta t\right)E_{\mathrm{LO}}$$

with electric fields *E*_SFG_ and *E*_LO_ of the SFG and LO. Both images were recorded simultaneously on the same CCD array, and their difference was calculated (using a paired pixel calibration measurement) to isolate the cross‐term response *S*_SFG_ ∝ *E*_SFG_ *E*_LO_.

The SFG images and spectra presented in this work were acquired interferometrically with 5 fs time steps from a mid‐IR – VIS delay time from −300 to 2500 fs. Each image was acquired for 1.5 s (unless otherwise noted) and averaged over 3 exposures. The full interferometric trace was recorded for azimuthal sample rotations in 15° steps ranging from 0 to 345°.

### Data Analysis

The obtained interferometric SFG images were first treated to remove any non‐zero offset in the interferograms, scaled by a Hanning window, and zero‐padded to double the number of time points. The result was then converted into spectral frequencies via a Fourier transformation and normalized in both amplitude and phase by a reference measurement of *z*‐cut α‐quartz (5 fs steps, −300 to 300 fs range, 1.5 s exposures, 3 averages). A singular value decomposition of the normalized data was then used to remove high spatial frequency imaging artifacts arising from the microscope geometry. These steps were implemented in the software Igor Pro.

The treated hyperspectral images for each sample rotation were then back‐rotated by the known rotation angle and spatially matched using the cross‐correlation of the simultaneously obtained linear reflection images of the LO, having removed the illumination envelope via 2D Fourier filtering. A complementary rotational matching approach based on machine learning produced similar results. The overlapped images were then subjected to a further rotational Fourier transformation to convert the azimuthal rotation angles into azimuthal frequencies,^[^ ^25^ ^]^ isolating the three‐fold frequency component that contains the crystallographic information of the monolayer hBN, which has D_3h_ symmetry (see Section S3, Supporting Information).

### Growth and Transfer of hBN Monolayers

hBN was grown via low‐pressure chemical vapor deposition (CVD) in a custom‐built hot‐walled system^[^ ^24^, ^28^ ^]^ on an iron (Fe) foil catalyst.^[^ ^53^ ^]^ Prior to growth, the Fe foil was etched in 0.2 m FeCl_3_ for 5 min to remove surface contaminants, then rinsed in water and dried before loading into the reactor. The Fe foil was first annealed at a temperature of 1050 °C for 30 min under 50 sccm of H_2_ (≈0.5 Torr) to reduce iron oxides and enlarge Fe grains. Under 50 sccm of H_2_ at 1050 °C, the solid precursor (ammonia‐borane, ≈3 mg) was heated to 90 °C upstream of the reactor, introducing B and N‐containing vapor species into the reactor to initiate hBN growth. After 30 min of growth, the precursor supply was shut off, and the system was cooled under H_2_. Figure S7 (Supporting Information) shows a scanning electron microscopy (SEM) image of hBN islands on the Fe catalyst. The image mostly showed triangular domains that had constant contrast, which was assigned to monolayers. Multilayers were instead expected to appear with a pyramidal structure,^[^ ^23^ ^]^ which was observed at a few locations.

hBN was transferred to fused silica (500 µm thick) via a sacrificial PMMA layer. A PMMA solution (4 wt.% in anisole) was first spin‐coated onto the hBN/Fe surface and allowed to dry. The Fe was then etched overnight by floating on a 0.2 m FeCl_3_ solution. After the Fe was completely dissolved, the PMMA/hBN was rinsed with 0.1 m HCl to remove FeCl_3_ and iron‐oxide residues before rising three times with DI water. The PMMA/hBN was then scooped onto the target fused silica, dried, and then heated to 90 °C to remove remaining water residues and ensure good adhesion. The PMMA layer was then dissolved overnight in acetone, the sample rinsed in isopropyl alcohol, and dried.

### AFM Imaging

The surface topography was characterized using an atomic force microscope (AFM, XE‐150 from Park Systems) in non‐contact mode with a scan rate of 0.23 Hz. A silicon tip (PPP‐NCHR) with 330 kHz tapping frequency and a set point of 12.7 nm was used. The images presented here are a cut‐out of a 45 × 45 µm scan with 1024 lines.

## Conflict of Interest

The authors declare no conflict of interest.

## Author Contributions

N.S.M. and A.P.F. contributed equally to this work. The project was conceived by A.P., M.T., and M.W. The SFG measurements were conducted by A.P.F., N.S.M., and B.J., and data were analyzed by A.P.F., N.S.M., and D.B. The samples were prepared by A.E.N., P.R.K., R.A.K., and J.D.C. AFM measurements were conducted by K.G.H. and N.S.M. DFT calculations were conducted by C.C. The data analysis was supported by D.B., H.H.H., C.S., and K.R. The manuscript was written by N.S.M. with contributions from all co‐authors.

## Supporting information



Supporting Information

Supplemental Video 1

Supplemental Video 2

## Data Availability

The data that support the findings of this study are openly available in Zenodo at https://doi.org/10.5281/zenodo.17339273, reference number 17339273.

## References

[1] C. R. Dean , A. F. Young , I. Meric , C. Lee , L. Wang , S. Sorgenfrei , K. Watanabe , T. Taniguchi , P. Kim , K. L. Shepard , J. Hone , Nat. Nanotechnol. 2010, 5, 722. 20729834 10.1038/nnano.2010.172

[2] S. Roy , X. Zhang , A. B. Puthirath , A. Meiyazhagan , S. Bhattacharyya , M. M. Rahman , G. Babu , S. Susarla , S. K. Saju , M. K. Tran , L. M. Sassi , M. A. S. R. Saadi , J. Lai , O. Sahin , S. M. Sajadi , B. Dharmarajan , D. Salpekar , N. Chakingal , A. Baburaj , X. Shuai , A. Adumbumkulath , K. A. Miller , J. M. Gayle , A. Ajnsztajn , T. Prasankumar , V. V. J. Harikrishnan , V. Ojha , H. Kannan , A. Z. Khater , Z. Zhu , et al., Adv. Mater. 2021, 33, 2101589. 10.1002/adma.202101589 34561916

[3] J. D. Caldwell , I. Aharonovich , G. Cassabois , J. H. Edgar , B. Gil , D. N. Basov , Nat. Rev. Mater. 2019, 4, 552.

[4] T. T. Tran , K. Bray , M. J. Ford , M. Toth , I. Aharonovich , Nat. Nanotechnol. 2016, 11, 37. 26501751 10.1038/nnano.2015.242

[5] J. D. Caldwell , A. V. Kretinin , Y. Chen , V. Giannini , M. M. Fogler , Y. Francescato , C. T. Ellis , J. G. Tischler , C. R. Woods , A. J. Giles , M. Hong , K. Watanabe , T. Taniguchi , S. A. Maier , K. S. Novoselov , Nat. Commun. 2014, 5, 5221. 25323633 10.1038/ncomms6221

[6] S. Dai , Z. Fei , Q. Ma , A. S. Rodin , M. Wagner , A. S. McLeod , M. K. Liu , W. Gannett , W. Regan , K. Watanabe , T. Taniguchi , M. Thiemens , G. Dominguez , A. H. C. Neto , A. Zettl , F. Keilmann , P. Jarillo‐Herrero , M. M. Fogler , D. N. Basov , Science 2014, 343, 1125. 24604197 10.1126/science.1246833

[7] P. Li , M. Lewin , A. V. Kretinin , J. D. Caldwell , K. S. Novoselov , T. Taniguchi , K. Watanabe , F. Gaussmann , T. Taubner , Nat. Commun. 2015, 6, 7507. 26112474 10.1038/ncomms8507 PMC4491815

[8] R. V. Gorbachev , I. Riaz , R. R. Nair , R. Jalil , L. Britnell , B. D. Belle , E. W. Hill , K. S. Novoselov , K. Watanabe , T. Taniguchi , A. K. Geim , P. Blake , Small 2011, 7, 465. 21360804 10.1002/smll.201001628

[9] J. Ling , X. Miao , Y. Sun , Y. Feng , L. Zhang , Z. Sun , M. Ji , ACS Nano 2019, 13, 14033. 31725258 10.1021/acsnano.9b06337

[10] E. Lin , M. Scherman , A. Pierret , B. Attal‐Tretout , A. Andrieux , L. Tailpied , T. Taniguchi , K. Watanabe , A. Loiseau , Opt. Lett. 2024, 49, 2329. 38691711 10.1364/OL.519571

[11] Y. Li , Y. Rao , K. F. Mak , Y. You , S. Wang , C. R. Dean , T. F. Heinz , Nano Lett. 2013, 13, 3329. 23718906 10.1021/nl401561r

[12] S. Kim , J. E. Fröch , A. Gardner , C. Li , I. Aharonovich , A. S. Solntsev , Opt. Lett. 2019, 44, 5792. 31774781 10.1364/OL.44.005792

[13] K. Yao , N. R. Finney , J. Zhang , S. L. Moore , L. Xian , N. Tancogne‐Dejean , F. Liu , J. Ardelean , X. Xu , D. Halbertal , K. Watanabe , T. Taniguchi , H. Ochoa , A. Asenjo‐Garcia , X. Zhu , D. N. Basov , A. Rubio , C. R. Dean , J. Hone , P. J. Schuck , Sci. Adv. 2021, 7, abe8691. 10.1126/sciadv.abe8691 PMC7929500 33658203

[14] T. Zhang , S. Qiao , H. Xue , Z. Wang , C. Yao , X. Wang , K. Feng , L.‐J. Li , D.‐K. Ki , Nano Lett. 2024, 24, 14774. 39527494 10.1021/acs.nanolett.4c04241

[15] L. Zhou , H. Fu , T. Lv , C. Wang , H. Gao , D. Li , L. Deng , W. Xiong , Nanomaterials 2020, 10, 2263. 33207552 10.3390/nano10112263 PMC7696749

[16] X. Yin , Z. Ye , D. A. Chenet , Y. Ye , K. O'Brien , J. C. Hone , X. Zhang , Science 2014, 344, 488. 24786072 10.1126/science.1250564

[17] W. Kim , J. Y. Ahn , J. Oh , J. H. Shim , S. Ryu , Nano Lett. 2020, 20, 8825. 33205983 10.1021/acs.nanolett.0c03763

[18] W. Huang , Y. Xiao , F. Xia , X. Chen , T. Zhai , Adv. Funct. Mater. 2024, 34, 2310726.

[19] Z. Xie , T. Zhao , X. Yu , J. Wang , Small 2024, 20, 2311621. 10.1002/smll.202311621 38618662

[20] J. E. Zimmermann , Y. D. Kim , J. C. Hone , U. Höfer , G. Mette , Nanoscale Horiz. 2020, 5, 1603. 33084712 10.1039/d0nh00396d

[21] T. Khan , B. John , R. Niemann , A. Paarmann , M. Wolf , M. Thämer , Opt. Express 2023, 31, 28792. 37710691 10.1364/OE.495903

[22] A. P. Fellows , B. John , M. Wolf , M. Thämer , Nat. Commun. 2024, 15, 3161. 38605056 10.1038/s41467-024-47573-1 PMC11009297

[23] A. E. Naclerio , P. R. Kidambi , Adv. Mater. 2023, 35, 2207374. 10.1002/adma.202207374 36329667

[24] A. E. Naclerio , P. Cheng , S. M. Hus , J. T. Diulus , M. Checa , I. Vlassiouk , W. H. Fissell , M. Coupin , J. Warner , L. Collins , A. Kolmakov , A.‐P. Li , P. R. Kidambi , Nano Lett. 2025, 25, 3221. 39950681 10.1021/acs.nanolett.4c05939 PMC11869279

[25] A. P. Fellows , B. John , M. Wolf , M. Thämer , J. Phys. Chem. Lett. 2024, 15, 10849. 39436358 10.1021/acs.jpclett.4c02679 PMC11533227

[26] M. Thämer , R. K. Campen , M. Wolf , Phys. Chem. Chem. Phys. 2018, 20, 25875. 30288514 10.1039/c8cp04239j

[27] H. Wang , T. Gao , W. Xiong , ACS Photonics 2017, 4, 1839.

[28] P. R. Kidambi , R. Blume , J. Kling , J. B. Wagner , C. Baehtz , R. S. Weatherup , R. Schloegl , B. C. Bayer , S. Hofmann , Chem. Mater. 2014, 26, 6380. 25673919 10.1021/cm502603n PMC4311958

[29] R. Niemann , S. Wasserroth , G. Lu , S. Gewinner , M. De Pas , W. Schöllkopf , J. D. Caldwell , M. Wolf , A. Paarmann , Appl. Phys. Lett. 2022, 120, 131102.

[30] M. Thämer , T. Garling , R. K. Campen , M. Wolf , J. Chem. Phys. 2019, 151, 064707. 10.1021/acs.jpca.9b09927 PMC6935974 31790247

[31] J. S. Ginsberg , M. M. Jadidi , J. Zhang , C. Y. Chen , N. Tancogne‐Dejean , S. H. Chae , G. N. Patwardhan , L. Xian , K. Watanabe , T. Taniguchi , J. Hone , A. Rubio , A. L. Gaeta , Nat. Commun. 2023, 14, 7685. 38001087 10.1038/s41467-023-43501-x PMC10673846

[32] W.‐T. Liu , Y. R. Shen , Phys. Rev. B 2008, 78, 024302.

[33] E. Roman , J. R. Yates , M. Veithen , D. Vanderbilt , I. Souza , Phys. Rev. B 2006, 74, 245204.

[34] R. Niemann , N. S. Mueller , S. Wasserroth , G. Lu , M. Wolf , J. D. Caldwell , A. Paarmann , Adv. Mater. 2024, 36, 2312507. 10.1002/adma.202312507 38895889

[35] F. Iyikanat , A. Konečná , F. J. García de Abajo , ACS Nano 2021, 15, 13415. 34310130 10.1021/acsnano.1c03775 PMC8388560

[36] W. Kim , G. Jeong , J. Oh , J. Kim , K. Watanabe , T. Taniguchi , S. Ryu , ACS Nano 2023, 17, 20580. 37801328 10.1021/acsnano.3c07428

[37] K. K. Kim , A. Hsu , X. Jia , S. M. Kim , Y. Shi , M. Hofmann , D. Nezich , J. F. Rodriguez‐Nieva , M. Dresselhaus , T. Palacios , J. Kong , Nano Lett. 2012, 12, 161. 22111957 10.1021/nl203249a

[38] Y. Liu , S. Bhowmick , B. I. Yakobson , Nano Lett. 2011, 11, 3113. 21732643 10.1021/nl2011142

[39] W. Auwärter , H. U. Suter , H. Sachdev , T. Greber , Chem. Mater. 2004, 16, 343.

[40] G. H. Ryu , H. J. Park , J. Ryou , J. Park , J. Lee , G. Kim , H. S. Shin , C. W. Bielawski , R. S. Ruoff , S. Hong , Z. Lee , Nanoscale 2015, 7, 10600. 25960354 10.1039/c5nr01473e

[41] H. Ma , C. Yang , B. Ni , Y. Li , S. Huang , W. Lin , Y. Zhang , ACS Nano 2025, 19, 16569. 40266006 10.1021/acsnano.5c00067

[42] L. Mennel , M. M. Furchi , S. Wachter , M. Paur , D. K. Polyushkin , T. Mueller , Nat. Commun. 2018, 9, 516. 29410470 10.1038/s41467-018-02830-y PMC5802795

[43] K. Frischwasser , K. Cohen , J. Kher‐Alden , S. Dolev , S. Tsesses , G. Bartal , Nat. Photonics 2021, 15, 442.

[44] L. Wang , J. Qi , W. Wei , M. Wu , Z. Zhang , X. Li , H. Sun , Q. Guo , M. Cao , Q. Wang , C. Zhao , Y. Sheng , Z. Liu , C. Liu , M. Wu , Z. Xu , W. Wang , H. Hong , P. Gao , M. Wu , Z.‐J. Wang , X. Xu , E. Wang , F. Ding , X. Zheng , K. Liu , X. Bai , Nature 2024, 629, 74. 38693415 10.1038/s41586-024-07286-3

[45] M. Zhao , Z. Ye , R. Suzuki , Y. Ye , H. Zhu , J. Xiao , Y. Wang , Y. Iwasa , X. Zhang , Light: Sci. Appl. 2016, 5, 16131. 10.1038/lsa.2016.131 PMC6059936 30167181

[46] G. Zograf , A. Y. Polyakov , M. Bancerek , T. J. Antosiewicz , B. Küçüköz , T. O. Shegai , Nat. Photonics 2024, 18, 751.

[47] C. Trovatello , C. Ferrante , B. Yang , J. Bajo , B. Braun , Z. H. Peng , X. Xu , P. K. Jenke , A. Ye , M. Delor , D. N. Basov , J. Park , P. Walther , C. R. Dean , L. A. Rozema , A. Marini , G. Cerullo , P. J. Schuck , Nat. Photonics 2025, 19, 291.

[48] R. Ma , H. Yan , Z. Zhou , Y. Yu , W. Wan , Opt. Lett. 2024, 49, 4565. 39146104 10.1364/OL.529620

[49] K. Yasuda , X. Wang , K. Watanabe , T. Taniguchi , P. Jarillo‐Herrero , Science 2021, 372, 1458. 10.1126/science.abd3230 34045323

[50] M. Vizner Stern , Y. Waschitz , W. Cao , I. Nevo , K. Watanabe , T. Taniguchi , E. Sela , M. Urbakh , O. Hod , M. Ben Shalom , Science 2021, 372, 1462. 10.1126/science.abe8177 34112727

[51] O. Minakova , C. Paiva , M. Frenzel , M. S. Spencer , J. M. Urban , C. Ringkamp , M. Wolf , G. Mussler , D. M. Juraschek , S. F. Maehrlein , arXiv Preprint 2025, arXiv:2503.11626. 10.1038/s41567-026-03274-8 PMC13423836 42539531

[52] M. Bonn , C. Hess , S. Funk , J. H. Miners , B. N. J. Persson , M. Wolf , G. Ertl , Phys. Rev. Lett. 2000, 84, 4653. 10990763 10.1103/PhysRevLett.84.4653

[53] P. Chaturvedi , A. E. Naclerio , S. Hus , I. Vlassiouk , N. Lavrik , M. Checa , L. Collins , A.‐P. Li , P. R. Kidambi , Adv. Mater. 2025, 11868. 10.1002/adma.202511868 PMC12862682 41312660

