# Supplementary material for: Full Crystallographic Imaging of Hexagonal Boron Nitride Monolayers with Phonon‐Enhanced Sum‐Frequency Microscopy
Source: Adv Mater. 2025 Nov 20;38(7):e10124. doi: 10.1002/adma.202510124 (PMC12862746; doi:10.1002/adma.202510124)
Supplement: Supplementary file 1 — Supporting Information [file ADMA-38-e10124-s003.pdf]

ADVANCED MATERIALS

Supporting Information

for *Adv. Mater.*, DOI 10.1002/adma.202510124

Full Crystallographic Imaging of Hexagonal Boron Nitride Monolayers with Phonon-Enhanced Sum-Frequency Microscopy

Niclas S. Mueller, Alexander P. Fellows, Ben John, Andrew E. Naclerio, Christian Carbogno, Katayoun Gharagozloo-Hubmann, Damián Baláž, Ryan A. Kowalski, Hendrik H. Heenen, Christoph Scheurer, Karsten Reuter, Joshua D. Caldwell, Martin Wolf, Piran R. Kidambi, Martin Thämer and Alexander Paarmann**

Supporting Information

Full Crystallographic Imaging of Hexagonal Boron Nitride Monolayers with Phonon-Enhanced Sum-Frequency Microscopy

Niclas S. Mueller[1,2†], Alexander P. Fellows[1†], Ben John[1], Andrew E. Naclerio[3], Christian Carbogno[4], Katayoun Gharagozloo-Hubmann[2], Damián Baláž[4], Ryan A. Kowalski[5,6], Hendrik H. Heenen[4], Christoph Scheurer[4], Karsten Reuter[4], Joshua D. Caldwell[5,6], Martin Wolf[1], Piran R. Kidambi[3,7], Martin Thämer[1,*], Alexander Paarmann[1,*]

[1] Department of Physical Chemistry, Fritz-Haber-Institute of the Max-Planck-Society, 14195, Berlin, Germany

[2] Department of Physics, Freie Universität Berlin, 14195, Berlin, Germany

[3] Department of Chemical and Biomolecular Engineering, Vanderbilt University, Nashville, TN, 37235, USA

[4] Theory Department, Fritz-Haber-Institute of the Max-Planck-Society, 14195, Berlin, Germany

[5] Department of Mechanical Engineering, Vanderbilt University, Nashville, TN, 37235, USA

[6] Interdisciplinary Materials Science Program, Vanderbilt University, Nashville, TN, 37235, USA

[7] Mechanical and Aerospace Engineering, University of Florida, Gainesville, FL, 32611, USA

[†] *contributed equally*

**corresponding authors (thaemer@fhi-berlin.mpg.de, alexander.paarmann@fhi-berlin.mpg.de)*

S1. Enhancement of SFG Signal by Phonon Resonance

As hBN does not have optical resonances in the visible spectral range, the frequency dependence of the SFG nonlinear susceptibility $\overleftrightarrow{\chi}^{(2)}$ only has resonances through its E' optical phonon in the mid-IR spectral range. Following Refs. 1,2, the SFG nonlinear susceptibility in the presence of an IR phonon resonance can be written as

$$\overleftrightarrow{\chi}^{(2)}(\omega_{\mathrm{IR}}) = \overleftrightarrow{\chi}^{(2)}_{\infty} + \sum_q \frac{\overleftrightarrow{A}_q}{\omega_q^2 - \omega_{\mathrm{IR}}^2 - i\gamma_q\omega_{\mathrm{IR}}}, \tag{S1}$$

with $\overleftrightarrow{\chi}^{(2)}_{\infty}$ the off-resonant high-frequency nonlinear susceptibility tensor, ω_q the IR phonon frequency of a phonon mode q, γ_q the phonon damping and $\overleftrightarrow{A}_q$ the amplitude of the $\chi^{(2)}$ phonon resonance. The amplitude tensor can be further written as[2,3]

$$A_{q,lmn} = \frac{\alpha_{q,lm} Z^*_{q,n}}{2V_{\mathrm{uc}}M}, \tag{S2}$$

with V_{uc} the volume of the primitive cell and M the reduced mass.

$$\alpha_{q,lm} = V_{\mathrm{uc}} \frac{\partial \chi^{(1)}_{\infty,lm}}{\partial u_q}\bigg|_{u=0} \tag{S3}$$

is the Raman polarizability tensor with $\chi^{(1)}_{\infty,lm}$ the elements of the non-resonant linear susceptibility tensor, and $u_q = u_{\mathrm{B},q} - u_{\mathrm{N},q}$ the relative displacement between the boron and nitrogen sublattices away from their equilibrium positions along the coordinate q.

$$Z^*_{q,n} = V_{\mathrm{uc}} \frac{\partial P_n}{\partial u_q}\bigg|_{u=0} \tag{S4}$$

is the Born effective charge, with $\mathbf{P}$ the macroscopic polarization.

In monolayer hBN we excite the E' mode, which has the Raman polarizability tensor[4]

$$\overleftrightarrow{\alpha}_b(E') = \begin{pmatrix} d & 0 \\ 0 & -d \end{pmatrix}, \qquad \overleftrightarrow{\alpha}_a(E') = \begin{pmatrix} 0 & d \\ d & 0 \end{pmatrix}, \tag{S5}$$

with the crystal a and b directions defined in Fig. S1a. This means that the Raman tensor has the non-vanishing components

$$\alpha_{b,bb} = -\alpha_{b,aa} = -\alpha_{a,ab} = -\alpha_{a,ba} = -d. \tag{S6}$$

Furthermore $Z^*_{q,n} \neq 0$ only when $q = n$, as the polarization $\mathbf{P} \parallel \mathbf{u}$. This together leads to the following non-vanishing components of the non-linear phonon amplitude

$$A_{b,bbb} = -A_{b,aab} = -A_{a,aba} = -A_{a,baa}, \tag{S7}$$

which matches the symmetry of the off-resonant D_{3h} second-order susceptibility $\chi^{(2)}_{bbb} = -\chi^{(2)}_{baa} = -\chi^{(2)}_{aab} = -\chi^{(2)}_{aba}$.[5] The polarization selection rules of SFG are therefore not changed by the IR excitation of the E' phonon and similar to off-resonant purely electronic SHG.

S2. DFT Calculations to Determine Sign of $\chi^{(2)}$ Tensor Elements

To investigate the response of hBN under SFG, we use density-functional theory (DFT), namely the implementation in the all-electron, local atomic orbitals based electronic-structure code *FHI-aims*.[6] The electronic-structure is described using a 16x16x1 grid of reciprocal-space **k**-points and 'tight' defaults for the numerical settings and local basis sets;[7] the Perdew-Burke-Enzerhof functional PBE[8] is used to model exchange and correlation at the semi-local level of theory and the non-local many-body dispersion approach is employed to incorporate van-der-Waals interactions.[9] First, the atomic and lattice degrees of freedom of the 2D-periodic BN sheet (see Fig. S1a, 100 Å of vacuum in z-direction) are fully relaxed,[10] whereby symmetry constraints[11] are used to retain the *P6₃/mmc* space group. In this equilibrium structure, vibrational frequencies at Γ are computed within the harmonic approximation using finite differences via *FHI-vibes*[12] and *phonopy*,[13] the respective IR and Raman intensities are obtained by additionally considering the finite-difference derivatives[14] of the (linear) polarization **P** and polarizability $\boldsymbol{\chi}$, respectively.

The polarization is defined as the dipole density

$$P_l = \frac{e}{V_{\mathrm{uc}}} \int_{V_{\mathrm{uc}}} r_l \, n(\mathbf{r}) \, dr^3 \quad \mathrm{mod}\, P_l^0, \tag{S8}$$

which is multi-valued up to an arbitrary multiple of the polarization quantum $\mathbf{P}_0$ under periodic conditions[15] and is here computed via a Berry phase formalism,[16] whereby the respective integral over reciprocal space is evaluated with 256 **k**-points along the polarization direction and 16 **k**-points perpendicular to it.

Conversely, the polarizability is defined as the derivative of the polarization upon the application of a vanishing, homogeneous electric field *E* along the Cartesian direction *m*

$$\chi_{lm} = \frac{V_{\mathrm{uc}}}{e} \left. \frac{\partial P_l}{\partial E_m} \right|_{E_m=0} = \int_{V_{\mathrm{uc}}} r_l \left. \frac{\partial n(\mathbf{r})}{\partial E_m} \right|_{E_m=0} dr^3. \tag{S9}$$

For its evaluation, the response of the density is required, which is here computed using the density-functional perturbation theory approach[17] presented in Ref. 18.

As shown in Fig. S1b, the computed IR response has a resonance at 1348 cm^{-1}, which belongs to the in-plane E' transverse optical (TO) phonon, as well as a weaker resonance at 800 cm^{-1}, which belongs to the out-of-plane A''$_2$ TO phonon (Fig. S1b, black). Only the in-plane E' TO phonon is also Raman-active and therefore contributes to the SFG response (Fig. S1b, blue).

To determine the sign of the $\chi^{(2)}$ tensor elements $A_{q,lmn}$ in Eq. (S2), we investigate how polarization and polarizability change around equilibrium for distortions along the E'-TO phonon mode with ω_{IR} = 1348 cm^{-1}. For the sake of clarity, we plot these data in Fig. S1c as function of changes in bond length for the nearest-neighbor BN pairs with the same *y*-position, see Fig. S1a for the definition of the employed coordinate system. Still, let us emphasize that not only one but all periodic B-N pairs are displaced along the E'-TO phonon eigenvector. From the data in Fig. S1c we find

$$Z^*_{b,b} = V_{\mathrm{uc}} \left. \frac{\partial P_b}{\partial u_b} \right|_{u=0} < 0, \quad \alpha_{b,bb} = V_{\mathrm{uc}} \left. \frac{\partial \chi^{(1)}_{\infty,bb}}{\partial u_b} \right|_{u=0} < 0, \tag{S10}$$

which leads to $A_{b,bbb} > 0$. Following Eq. (S7), this determines the sign of all $\chi^{(2)}$ tensor elements. At $\omega_{\mathrm{IR}} = \omega_{\mathrm{TO}}$ we find $\mathrm{Im}\left[\chi^{(2)}_{bbb}\right] > 0$, see Eq. (S1), which lets us determine the full crystal orientation of hBN from SFG measurements, see SI Section S3.

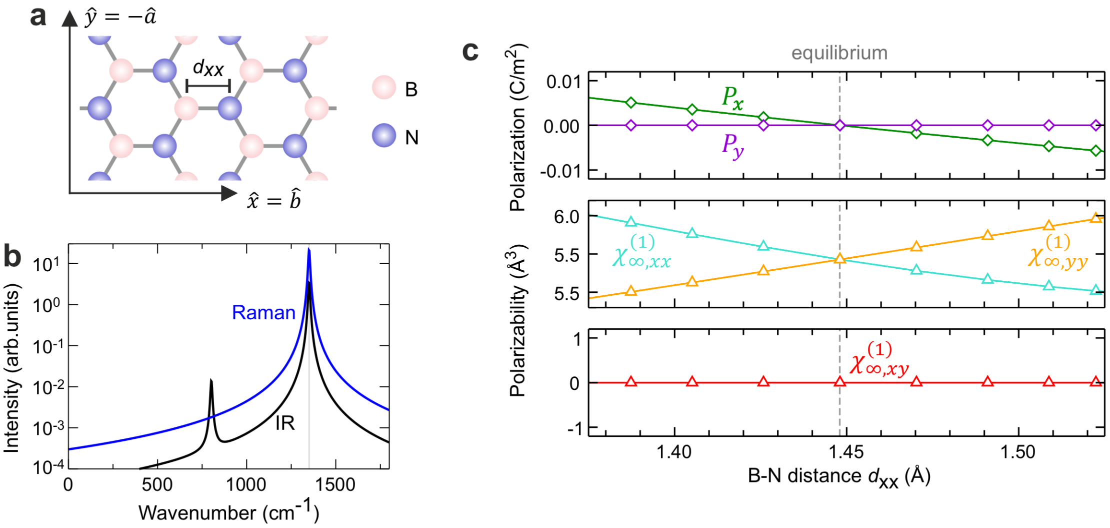

Figure S1. (a) hBN crystal structure with crystal $\hat{b}$ axis chosen along B-N arm-chair direction ($\hat{b}$ = $\hat{x}$ here). (b) IR (black) and Raman (blue) spectra calculated with DFT for the hBN monolayer. (c) IR polarization P_n of lattice (top) and electronic polarizability $\chi^{(1)}_{\infty,lm}$ (bottom) for different B-N bond distances d_{xx}. The computed equilibrium bond distance of 1.448 Å is indicated by the grey dashed line.

S3. Determining the Crystal Orientation of Monolayer hBN from Phase-Resolved SFG

Monolayer hBN belongs to the D_{3h} point group, with the non-vanishing second-order susceptibility tensor components $\chi^{(2)}_{bbb} = -\chi^{(2)}_{baa} = -\chi^{(2)}_{aab} = -\chi^{(2)}_{aba}$.[5] In the crystal coordinate system a, b, c, the arm-chair crystal direction is aligned with $\hat{b}$ (Fig. S1a). $\hat{b}$ is chosen here to point along the B-N direction, i.e. the N-B direction is along $-\hat{b}$. In order to account for an arbitrary orientation of the hBN lattice, we use a crystal (a, b, c) to lab (x, y, z) frame transformation[1]

$$\chi^{(2)}_{ijk} = \sum_{lmn} \chi^{(2)}_{lmn} (\hat{\imath} \cdot \hat{l})(\hat{\jmath} \cdot \hat{m})(\hat{k} \cdot \hat{n}), \tag{S11}$$

with $(\hat{\imath}, \hat{\jmath}, \hat{k})$ and $(\hat{l}, \hat{m}, \hat{n})$ unit vectors defining the lab and crystal frame respectively. We assume that hBN is placed in the *xy* plane. A rotation about $\hat{z}$ by an angle φ corresponds to the coordinate transformation $\hat{x} = \hat{a}\sin\varphi + \hat{b}\cos\varphi$, $\hat{y} = -\hat{a}\cos\varphi + \hat{b}\sin\varphi$, and $\hat{c} = \hat{z}$, which can be described by the rotation matrix (Fig. S2a)

$$\begin{pmatrix} \hat{x} \\ \hat{y} \end{pmatrix} = \overleftrightarrow{R} \begin{pmatrix} \hat{a} \\ \hat{b} \end{pmatrix} = \begin{pmatrix} \sin\varphi & \cos\varphi \\ -\cos\varphi & \sin\varphi \end{pmatrix} \begin{pmatrix} \hat{a} \\ \hat{b} \end{pmatrix}. \tag{S12}$$

The nonlinear SFG polarization is given by

$$P_{\mathrm{SFG},i} = \epsilon_0 \sum_{jk} \chi^{(2)}_{ijk} E_{\mathrm{VIS},j} E_{\mathrm{IR},k}. \tag{S13}$$

In our experiments we used p-polarized light with $\mathbf{E}_{\mathrm{IR}} = E_{\mathrm{IR}}\{\cos\vartheta, 0, \sin\vartheta\}$ and $\mathbf{E}_{\mathrm{VIS}} = E_{\mathrm{VIS}}\{\cos\vartheta, 0, \sin\vartheta\}$, with ϑ the incidence angle with respect to the z axis. Applying the coordinate transformation and considering all second-order tensor components, we obtain

$$\mathbf{P}_{\mathrm{SFG}} = \chi^{(2)}_{bbb} E_{\mathrm{VIS},x} E_{\mathrm{IR},x} \cos^2\vartheta \begin{pmatrix} \cos(3\varphi) \\ \sin(3\varphi) \\ 0 \end{pmatrix}. \tag{S14}$$

In the experiments we detect the radiated electric field S_{SFG} that also depends on Fresnel factors for light in- and outcoupling, the interference with the local oscillator (LO), as well as the sensitivity of the setup, see SI Section S4. In the experiments the polarization of the local oscillator (LO) is set to 's' such that the *y*-component of the SFG signal $S_{\mathrm{SFG},y}$ is suppressed by the balanced imaging and only the *x*-component $S_{\mathrm{SFG},x} \propto \cos(3\varphi)$ is detected.[19]

From DFT simulations and Fresnel factors, see SI Sections S2 and S4, we expect $\mathrm{Im}[S_{\mathrm{SFG},x}(\varphi = 0)] > 0$. The sample is rotated clockwise with a rotation stage by φ_{rot} ($\varphi_{\mathrm{rot}} > 0$ for clockwise rotation). To distinguish this rotation from the crystal orientation, we define $\varphi = \varphi_{\mathrm{cryst}} - \varphi_{\mathrm{rot}}$, where φ_{cryst} is the angle between the crystal b axis (arm-chair crystal direction along B-N) and the lab frame positive x axis at $\varphi_{\mathrm{rot}} = 0$. Figure S2b shows the calculated φ_{rot}-dependence of $\mathrm{Im}[S_{\mathrm{SFG},x}(\varphi_{\mathrm{rot}})]$ for $\varphi_{\mathrm{cryst}} = 70°$. The positive maxima of $\mathrm{Im}[S_{\mathrm{SFG},x}(\varphi_{\mathrm{rot}})]$ occur at $\varphi_{\mathrm{rot}} = \varphi_{\mathrm{cryst}} + 2n\pi/3$, with $n = 0,1,2$, and can thus be used to determine the B-N arm-chair crystal direction of hBN.

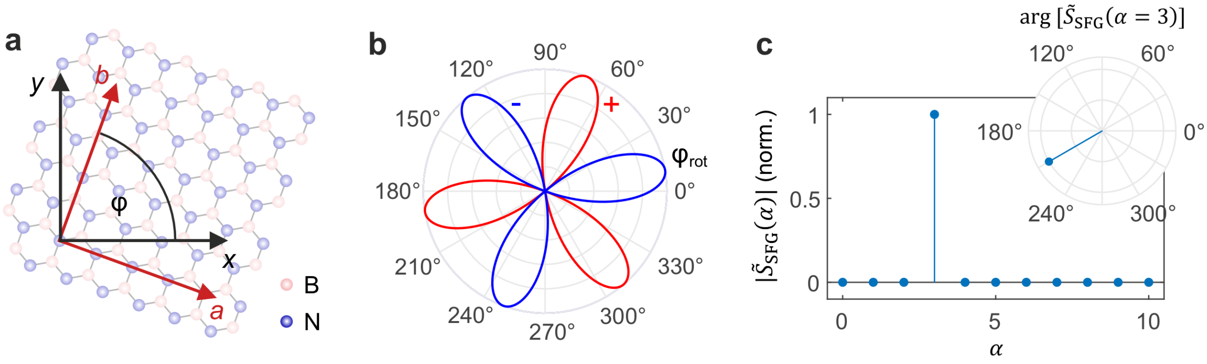

Figure S2. (a) Crystal frame (red) *vs* lab frame (black). The crystal b axis is along the B-N arm-chair direction. φ measures the angle between the x axis and the crystal b axis. (b) Calculated polar plot of $\mathrm{Im}[S_{\mathrm{SFG},x}(\varphi_{\mathrm{rot}})]$ for $\varphi_{\mathrm{cryst}} = 70°$, as shown in (a). Red indicates positive and blue negative sign. (c) Fourier transform $\tilde{S}_{\mathrm{SFG}}(\alpha)$ of the rotation dependence of $\mathrm{Im}[S_{\mathrm{SFG},x}(\varphi_{\mathrm{rot}})]$ in (b) with magnitude giving three-fold symmetry and phase $3\varphi_{\mathrm{cryst}}$. α gives the order of the rotational symmetry.

In the experiments, the crystal orientation is determined for each image pixel by a Fourier transformation of $\mathrm{Im}[S_{\mathrm{SFG},x}(\varphi_{\mathrm{rot}})]$ (see also Refs. 20,21)

$$\tilde{S}_{\mathrm{SFG}}(\alpha) = \frac{1}{\sqrt{2\pi}}\int_{-\infty}^{\infty} d\varphi_{\mathrm{rot}}\, \mathrm{Im}[S_{\mathrm{SFG},x}(\varphi_{\mathrm{rot}})]\, e^{i\alpha\varphi_{\mathrm{rot}}} \tag{S15}$$

$$\propto \mathrm{Im}[\chi^{(2)}_{bbb} E_{\mathrm{VIS},x} E_{\mathrm{IR},x}] \cos^2\vartheta \frac{1}{\sqrt{2\pi}}\int_{-\infty}^{\infty} d\varphi_{\mathrm{rot}} \cos[3(\varphi_{\mathrm{cryst}} - \varphi_{\mathrm{rot}})]\, e^{i\alpha\varphi_{\mathrm{rot}}}$$

$$= \mathrm{Im}\left[\chi^{(2)}_{bbb} E_{\mathrm{VIS},x} E_{\mathrm{IR},x}\right] \cos^2\vartheta \sqrt{\frac{\pi}{2}}\left[\delta(\alpha-3)e^{3i\varphi_{\mathrm{cryst}}} + \delta(\alpha+3)e^{-3i\varphi_{\mathrm{cryst}}}\right],$$

where α gives the order of the rotational symmetry. The magnitude of the rotational Fourier transformation gives the 3-fold symmetry of the crystal structure (neglecting components with $\alpha < 0$)

$$|\tilde{S}_{\mathrm{SFG}}(\alpha)| \propto \delta(\alpha - 3) \tag{S16}$$

and the rotational phase the crystal orientation

$$\arg[\tilde{S}_{\mathrm{SFG}}(\alpha = 3)] = 3\varphi_{\mathrm{cryst}}. \tag{S17}$$

Figure S2c shows the magnitude and phase of $\tilde{S}_{\mathrm{SFG}}(\alpha)$ for the polar plot in Fig. S2b, when sampled in discrete steps of $\Delta\varphi_{\mathrm{rot}} = 15°$ as in the experiments. Both, crystal symmetry α and crystal orientation φ_{cryst} can be determined. In the main text, we use the nomenclature $\tilde{S}_{\mathrm{SFG}}(\alpha = 3) \equiv \tilde{S}_{\mathrm{SFG,3f}}$ for the rotational SFG amplitude with 3-fold symmetry.

S4. Determining the $\chi^{(2)}$ SFG Magnitude and Phase of hBN from Experiments

We use z-cut α-quartz (Q) as a reference to determine the absolute magnitude and phase of the experimental hBN $\chi^{(2)}$ SFG response.[22] In the ppp-polarization combination, we probe the $\chi^{(2)}_{aaa,\mathrm{Q}}$ tensor component of quartz,[23] which is known to be $\chi^{(2)}_{aaa,\mathrm{Q}} \approx 0.6\ \mathrm{pm/V}$, if off-resonant.[24] Following Ref. 25, the magnitude of the nonlinear susceptibility of hBN can be estimated by referencing with quartz:

$$\chi^{(2)}_{\mathrm{eff,hBN}} = \frac{L^{\mathrm{Q}}_{xx}(\omega_{\mathrm{SFG}})L^{\mathrm{Q}}_{xx}(\omega_{\mathrm{VIS}})L^{\mathrm{Q}}_{xx}(\omega_{\mathrm{IR}})}{L^{\mathrm{hBN}}_{xx}(\omega_{\mathrm{SFG}})L^{\mathrm{hBN}}_{xx}(\omega_{\mathrm{VIS}})L^{\mathrm{hBN}}_{xx}(\omega_{\mathrm{IR}})} \frac{r_{\mathrm{Q}}(\omega_{\mathrm{SFG}})}{r_{\mathrm{hBN}}(\omega_{\mathrm{SFG}})} \frac{i\chi^{(2)}_{aaa,\mathrm{Q}}}{|\Delta k_z|} \frac{S_{\mathrm{hBN}}}{S_{\mathrm{Q}}}, \tag{S18}$$

with the Fresnel factors $L^{Q}_{xx}(\omega_j)$ across the air-quartz interface at frequency ω_j, the complex-valued SFG amplitude signals S_{hBN} and S_{Q} of hBN and quartz, and the wavevector mismatch $\Delta k_z = k_z(\omega_{\mathrm{SFG}}) + k_z(\omega_{\mathrm{VIS}}) + k_z(\omega_{\mathrm{IR}})$ in quartz in the reflection geometry. In our experiments, the hBN monolayers are placed on a fused silica substrate. As hBN is an atomically thin 2D polarizable sheet, we calculate the Fresnel factors $L^{\mathrm{hBN}}_{xx}(\omega_j)$ by dividing the total electric field amplitude $E_{\mathrm{air-SiO2}}(\omega_j)$ at the air-fused silica interface by the amplitude of the incident electric field in air. We do not use a three-layer geometry air-hBN-SiO_2 treating hBN as a bulk medium, where Fresnel factors are calculated from air into hBN, as this approach is known to overestimate $\chi^{(2)}_{\mathrm{eff}}$ for atomically thin 2D materials.[26] In addition we include reflection coefficients $r_{\mathrm{Q}}(\omega_{\mathrm{SFG}})$ and $r_{\mathrm{hBN}}(\omega_{\mathrm{SFG}})$ for the s-polarized local oscillator (LO) beams that are used for heterodyne detection.

We determine the Fresnel factors and wavevector mismatch with transfer matrix calculations accounting for the optical anisotropy of the materials.[27] We use the IR dielectric functions from Ref. 28 for α-quartz, and Ref. 29 for fused silica. Figure S3 shows the multiplied Fresnel factors $L^{m}_{xx}(\omega_{\mathrm{SFG}})L^{m}_{xx}(\omega_{\mathrm{VIS}})L^{m}_{xx}(\omega_{\mathrm{IR}})$ for the air-quartz (Fig. S3a) and the air-fused silica interfaces (Fig. S3b). For frequencies >1300 cm^{-1} the Fresnel factors are real-valued and ~0.72 and ~0.78 respectively. At lower frequencies the Fresnel factors are instead modulated because of IR phonon resonances in quartz and fused silica. In the spectral range 1200-1300 cm^{-1}, the modulations occur at the zero-crossings of the dielectric functions of quartz and fused silica at ≈1240 cm^{-1} and 1264 cm^{-1}. Overall, the ratio of the Fresnel correction factors in Eq. (S18) is almost constant, real-valued (~0.92 at $\omega_{\mathrm{IR}} = \omega_{\mathrm{TO}}$) at ω_{IR}>1300 cm^{-1} vs strongly modulated for ω_{IR}<1300 cm^{-1} (Fig. S3c), which explains the side peak in the experimental SFG spectrum in Fig. 2b, main text. Figure S3d shows the wavevector mismatch $|\Delta k_z|$ in quartz, giving the coherence length of the SFG signal, which shows that a depth of ~36 nm is probed in the SFG reference measurements on α-quartz. In addition, we obtain $r_{\mathrm{Q}}(\omega_{\mathrm{SFG}})/r_{\mathrm{hBN}}(\omega_{\mathrm{SFG}})$~1.13.

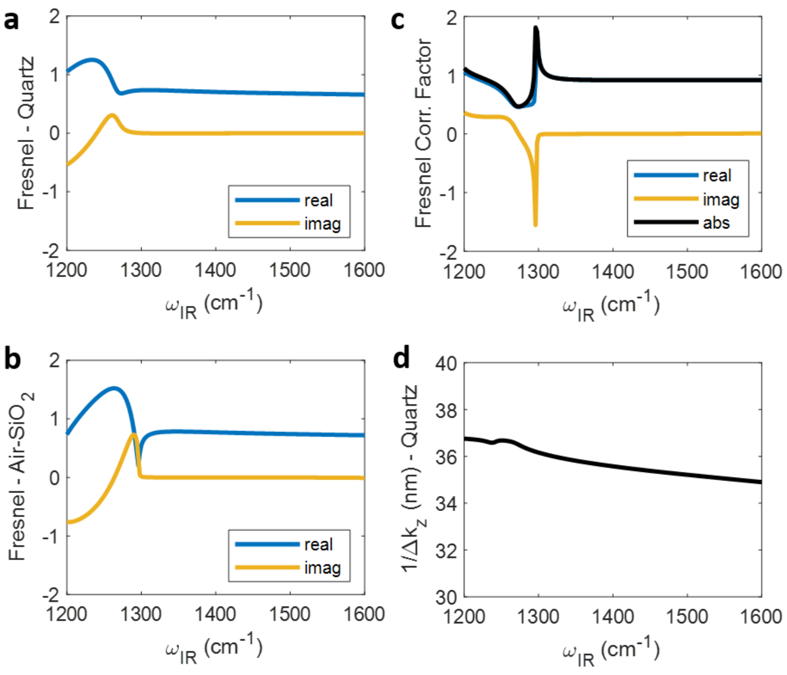

Figure S3. (a) Fresnel factors $L_{xx}^{\text{Q}}(\omega_{\text{SFG}})L_{xx}^{\text{Q}}(\omega_{\text{VIS}})L_{xx}^{\text{Q}}(\omega_{\text{IR}})$ of air - *z*-cut α-quartz interface and (b) of air - fused silica interface $L_{xx}^{\text{hBN}}(\omega_{\text{SFG}})L_{xx}^{\text{hBN}}(\omega_{\text{VIS}})L_{xx}^{\text{hBN}}(\omega_{\text{IR}})$ as function of ω_{IR}. (c) Total Fresnel correction factor (a)/(b) in Eq. (S18). (d) Inverse of wavevector mismatch $\Delta k_z = |k_z(\omega_{\text{SFG}}) + k_z(\omega_{\text{VIS}}) + k_z(\omega_{\text{IR}})|$ vs ω_{IR}.

We measured an SFG signal of hBN for ω_{IR} = 1368 cm^{-1} at the phonon resonance that is ~10 times larger than that of α-quartz, i.e. $|S_{\text{hBN}}/S_{\text{Q}}| \approx 8.5$. Using Eq. (S18), this corresponds to an effective surface susceptibility of

$$\chi^{(2)}_{\text{eff,hBN,SFG}}(\omega_{\text{IR}} = 1368\ \text{cm}^{-1}) \approx 1.9 \cdot 10^{-19}\ \text{m}^2/\text{V}. \tag{S19}$$

When dividing by the thickness of hBN d_{hBN} = 0.33 nm (interlayer separation in a bulk crystal), this corresponds to an associated bulk susceptibility of

$$\chi^{(2)}_{\text{hBN,SFG}}(\omega_{\text{IR}} = 1368\ \text{cm}^{-1}) \approx 580\ \text{pm/V}, \tag{S20}$$

which is 970x larger than that of α-quartz.

In a previous experiment Li et al. measured the off-resonant SHG intensity of monolayer hBN and *z*-cut α-quartz using λ_{exc} = 810 nm excitation at normal incidence and obtained[5]

$$\chi^{(2)}_{\text{eff,hBN,SHG}} \approx 6.9 \cdot 10^{-21} \text{m}^2/\text{V} \qquad \rightarrow \qquad \chi^{(2)}_{\text{hBN,SHG}} \approx 20.8\ \text{pm/V}. \tag{S21}$$

The phonon-enhanced resonant $\chi^{(2)}_{\text{hBN,SFG}}$ is thus 28x larger than the off-resonant $\chi^{(2)}_{\text{hBN,SHG}}$. Assuming that the off-resonant $\chi^{(2)}_{\text{hBN}}$ for SFG and SHG are the same, this corresponds to a 790-fold enhancement of $\left|\chi^{(2)}_{\text{hBN}}\right|^2$ by the phonon resonance, Fig. 2c main text.

S5. Further Supplementary Figures

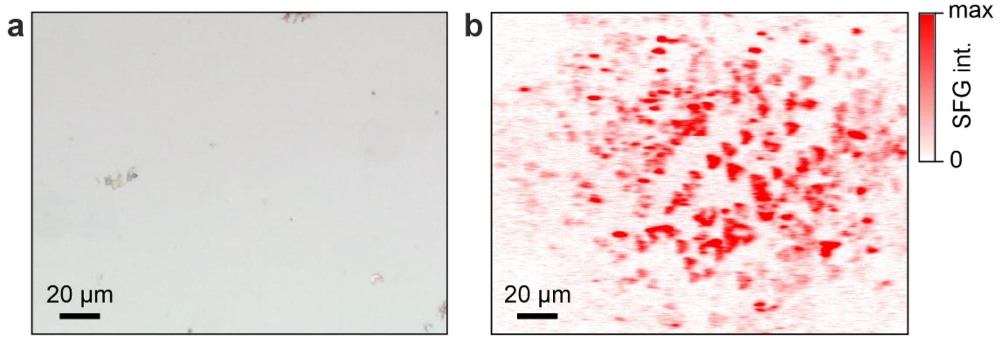

Figure S4. (a) Linear bright-field microscope image in reflection of hBN monolayers on a fused silica substrate. Only contaminations are visible. (b) SFG microscope image of same sample area as in (a) recorded in 0.5 s, only using IR and VIS lasers without heterodyning.

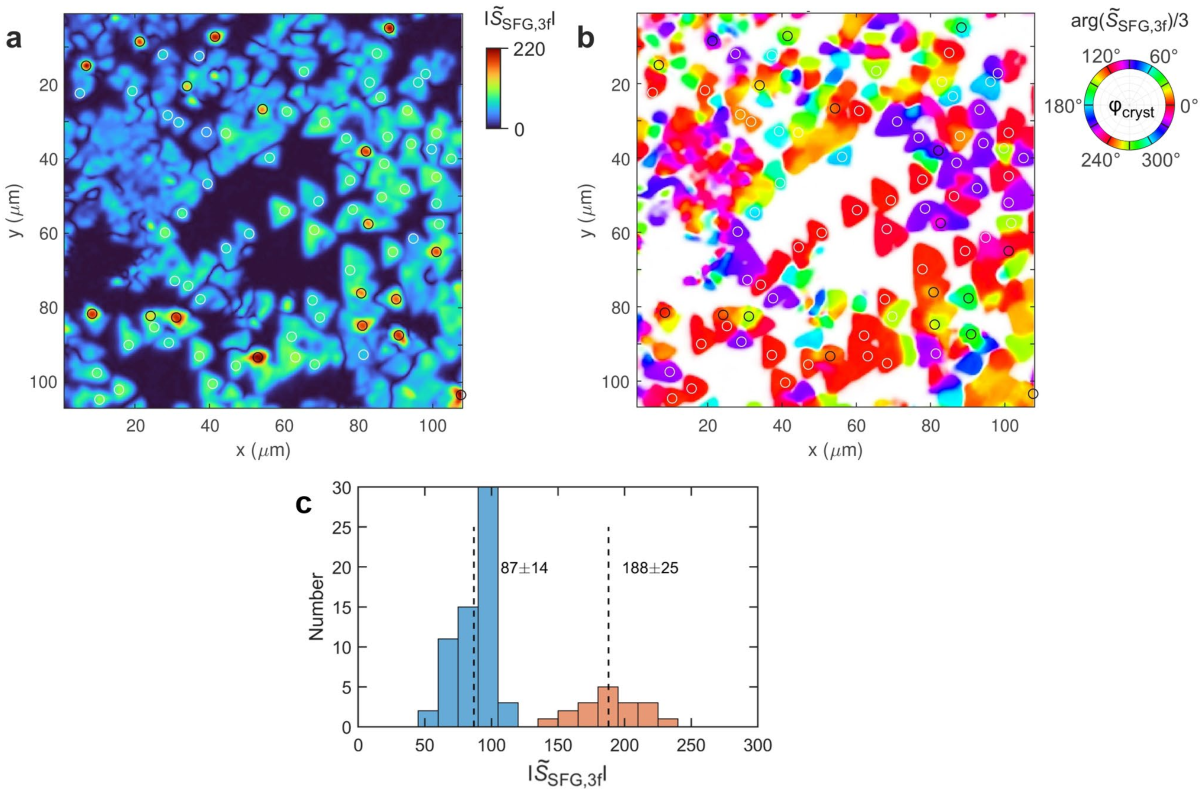

Figure S5. (a) Rotational amplitude $|\tilde{E}_{\mathrm{SFG,3f}}|$ and (b) phase $\arg(\tilde{E}_{\mathrm{SFG,3f}})/3$ of the SFG signal with 3-fold rotational symmetry, as in Fig. 3c,e main text, but for a larger sample area, which is accessible within the field of view when azimuthally rotating the sample. (c) Distribution of maximum values of the rotational amplitude $|\tilde{E}_{\mathrm{SFG,3f}}|$ in monolayer (blue) and multilayer (red) patches, indicated as white and black circles, respectively, in (a) and (b). Only patches with spatially homogeneous phase were considered for the statistical analysis.

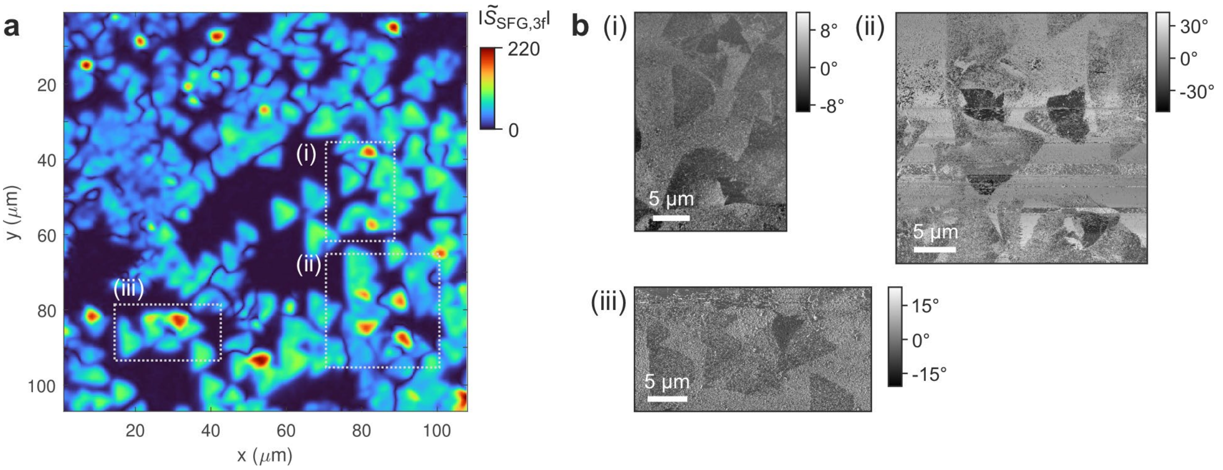

Figure S6. (a) 3-Fold rotational SFG amplitude $|\tilde{E}_{\mathrm{SFG,3f}}|$ with dashed boxes highlighting areas that were characterized by AFM. (b) AFM phase images of areas (i)-(iii) in (a). Bright spots in (a) correspond to darker areas in (b) that are likely bi- and few-layers.

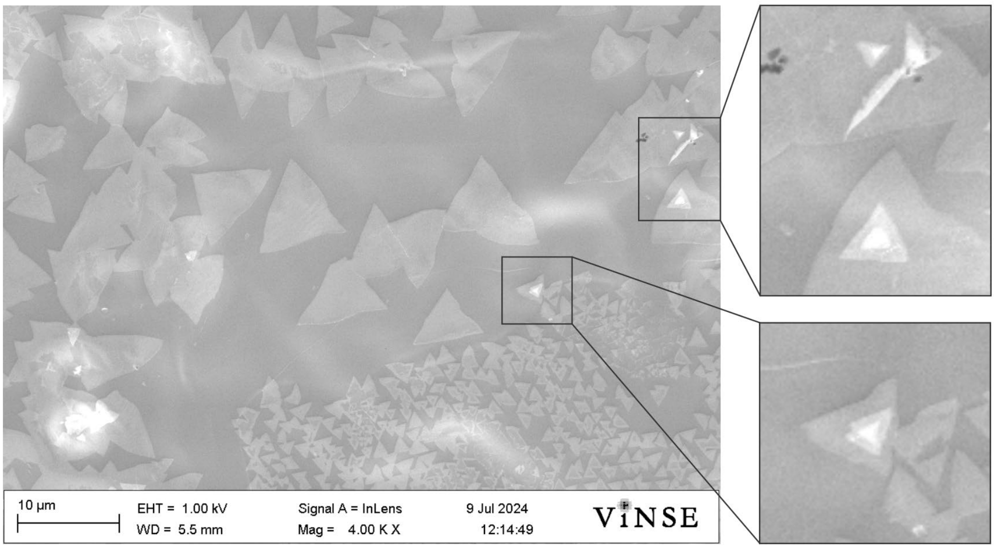

Figure S7. Scanning electron microscopy image of hBN monolayer islands on Fe catalyst after CVD growth. Boxes and magnified images on the right highlight the growth of additional layers at a few locations. Images are collected with a Zeiss Merlin scanning electron microscope with Gemini II column operated at 2 keV acceleration voltage.

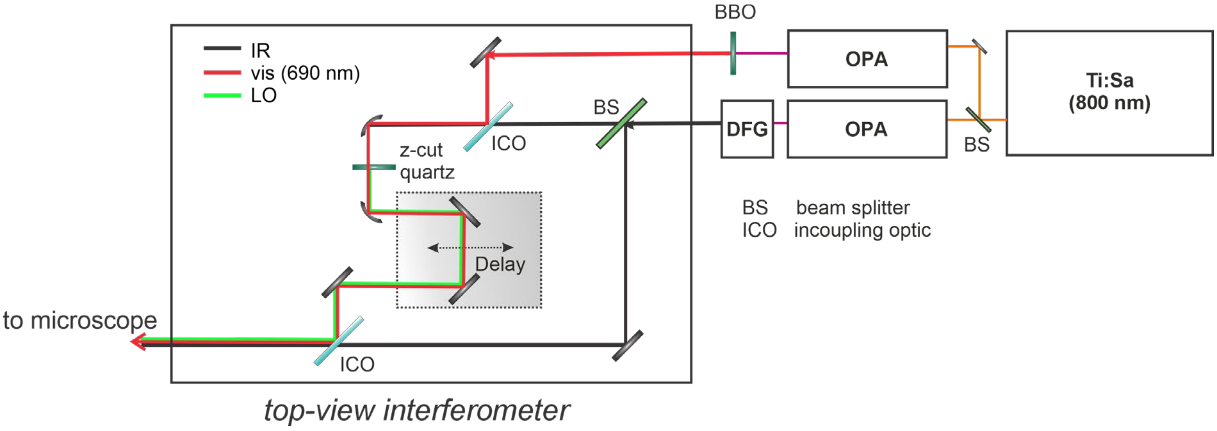

Figure S8. Schematic of the laser system and SFG interferometer employed for heterodyned SFG microscopy. An amplified Ti:Sa laser (Coherent Astrella) drives two commercial OPAs (Light Conversion Topas Prime), one for generating the visible upconversion beam, and one for difference-frequency generation between its signal and idler beams to provide the IR pulses. Subsequently, the local oscillator is generated in a *z*-cut quartz crystal, and after a delay line for LO and VIS pulses, all beams are recombined collinearly and guided into the microscope.

References

1 Liu, W.-T. & Shen, Y. R. Sum-frequency phonon spectroscopy on alpha-quartz. *Physical Review B* **78**, 024302, doi:10.1103/PhysRevB.78.024302 (2008).

2 Roman, E., Yates, J. R., Veithen, M., Vanderbilt, D. & Souza, I. Ab initio study of the nonlinear optics of III-V semiconductors in the terahertz regime. *Physical Review B* **74**, 245204, doi:10.1103/PhysRevB.74.245204 (2006).

3 Flytzanis, C. Infrared Dispersion of Second-Order Electric Susceptibilities in Semiconducting Compounds. *Physical Review B* **6**, 1264-1290, doi:10.1103/PhysRevB.6.1264 (1972).

4 Huang, M. *et al.* Phonon softening and crystallographic orientation of strained graphene studied by Raman spectroscopy. *Proceedings of the National Academy of Sciences* **106**, 7304-7308, doi:10.1073/pnas.0811754106 (2009).

5 Li, Y. *et al.* Probing Symmetry Properties of Few-Layer MoS2 and h-BN by Optical Second-Harmonic Generation. *Nano Letters* **13**, 3329-3333, doi:10.1021/nl401561r (2013).

6 Blum, V. *et al.* Ab initio molecular simulations with numeric atom-centered orbitals. *Computer Physics Communications* **180**, 2175-2196, doi:10.1016/j.cpc.2009.06.022 (2009).

7 Carbogno, C. *et al.* Numerical quality control for DFT-based materials databases. *npj Computational Materials* **8**, 69, doi:10.1038/s41524-022-00744-4 (2022).

8 Perdew, J. P., Burke, K. & Ernzerhof, M. Generalized Gradient Approximation Made Simple [Phys. Rev. Lett. 77, 3865 (1996)]. *Physical Review Letters* **78**, 1396-1396, doi:10.1103/PhysRevLett.78.1396 (1997).

9 Hermann, J. & Tkatchenko, A. Density Functional Model for van der Waals Interactions: Unifying Many-Body Atomic Approaches with Nonlocal Functionals. *Physical Review Letters* **124**, 146401, doi:10.1103/PhysRevLett.124.146401 (2020).

10 Knuth, F., Carbogno, C., Atalla, V., Blum, V. & Scheffler, M. All-electron formalism for total energy strain derivatives and stress tensor components for numeric atom-centered orbitals. *Computer Physics Communications* **190**, 33-50, doi:10.1016/j.cpc.2015.01.003 (2015).
11 Lenz, M.-O. *et al.* Parametrically constrained geometry relaxations for high-throughput materials science. *npj Computational Materials* **5**, 123, doi:10.1038/s41524-019-0254-4 (2019).
12 Knoop, F., Purcell, T. A. R., Scheffler, M. & Carbogno, C. FHI-vibes: Ab Initio Vibrational Simulations. *Journal of Open Source Software* **5**, 2671, doi:10.21105/joss.02671 (2020).
13 Togo, A., Chaput, L., Tadano, T. & Tanaka, I. Implementation strategies in phonopy and phono3py. *Journal of Physics: Condensed Matter* **35**, 353001, doi:10.1088/1361-648X/acd831 (2023).
14 Akkoush, A., Litman, Y. & Rossi, M. A Hybrid-Density Functional Theory Study of Intrinsic Point Defects in MX2 (M = Mo, W; X = S, Se) Monolayers. *physica status solidi (a)* **221**, 2300180, doi:10.1002/pssa.202300180 (2024).
15 King-Smith, R. D. & Vanderbilt, D. Theory of polarization of crystalline solids. *Physical Review B* **47**, 1651-1654, doi:10.1103/PhysRevB.47.1651 (1993).
16 Carbogno, C. *et al.* Polarisation, Born Effective Charges, and Topological Invariants via a Berry-Phase Approach. *arXiv [cond-mat.mtrl-sci]* (2025).
17 Baroni, S., de Gironcoli, S., Dal Corso, A. & Giannozzi, P. Phonons and related crystal properties from density-functional perturbation theory. *Reviews of Modern Physics* **73**, 515-562, doi:10.1103/RevModPhys.73.515 (2001).
18 Shang, H. *et al.* All-electron, real-space perturbation theory for homogeneous electric fields: theory, implementation, and application within DFT. *New Journal of Physics* **20**, 073040, doi:10.1088/1367-2630/aace6d (2018).
19 Khan, T. *et al.* Compact oblique-incidence nonlinear widefield microscopy with paired-pixel balanced imaging. *Opt. Express* **31**, 28792-28804, doi:10.1364/OE.495903 (2023).
20 Fellows, A. P., John, B., Wolf, M. & Thämer, M. Spiral packing and chiral selectivity in model membranes probed by phase-resolved sum-frequency generation microscopy. *Nature Communications* **15**, 3161, doi:10.1038/s41467-024-47573-1 (2024).
21 Fellows, A. P., John, B., Wolf, M. & Thämer, M. Extracting the Heterogeneous 3D Structure of Molecular Films Using Higher Dimensional SFG Microscopy. *The Journal of Physical Chemistry Letters* **15**, 10849-10857, doi:10.1021/acs.jpclett.4c02679 (2024).
22 Hu, X.-H., Wei, F., Wang, H. & Wang, H.-F. α-Quartz Crystal as Absolute Intensity and Phase Standard in Sum-Frequency Generation Vibrational Spectroscopy. *The Journal of Physical Chemistry C* **123**, 15071-15086, doi:10.1021/acs.jpcc.9b03202 (2019).
23 Thämer, M., Garling, T., Campen, R. K. & Wolf, M. Quantitative determination of the nonlinear bulk and surface response from alpha-quartz using phase sensitive SFG spectroscopy. *The Journal of Chemical Physics* **151**, doi:10.1063/1.5109868 (2019).
24 Boyd, R. W. *Nonlinear Optics (Third Edition)*. (Academic Press, 2008).
25 Fellows, A. P. *et al.* Sum-Frequency Generation Spectroscopy of Aqueous Interfaces: The Role of Depth and Its Impact on Spectral Interpretation. *The Journal of Physical Chemistry C* **128**, 20733-20750, doi:10.1021/acs.jpcc.4c06650 (2024).
26 Clark, D. J. *et al.* Strong optical nonlinearity of CVD-grown MoS2 monolayer as probed by wavelength-dependent second-harmonic generation. *Physical Review B* **90**, 121409, doi:10.1103/PhysRevB.90.121409 (2014).
27 Passler, N. C. & Paarmann, A. Generalized 4 × 4 matrix formalism for light propagation in anisotropic stratified media: study of surface phonon polaritons in polar dielectric heterostructures. *J. Opt. Soc. Am. B* **34**, 2128-2139, doi:10.1364/JOSAB.34.002128 (2017).
28 Spitzer, W. G. & Kleinman, D. A. Infrared Lattice Bands of Quartz. *Physical Review* **121**, 1324-1335, doi:10.1103/PhysRev.121.1324 (1961).
29 Cataldo, G., Wollack, E. J., Brown, A. D. & Miller, K. H. Infrared dielectric properties of low-stress silicon oxide. *Opt. Lett.* **41**, 1364-1367, doi:10.1364/OL.41.001364 (2016).
